# Molecular Epidemiology of HIV-1 Subtype B Reveals Heterogeneous Transmission Risk: Implications for Intervention and Control

**DOI:** 10.1093/infdis/jiy044

**Published:** 2018-02-26

**Authors:** Erik M Volz, Stephane Le Vu, Oliver Ratmann, Anna Tostevin, David Dunn, Chloe Orkin, Siobhan O’Shea, Valerie Delpech, Alison Brown, Noel Gill, Christophe Fraser, UK HIV Drug Resistance Database

**Affiliations:** 1 Department of Infectious Disease Epidemiology and the National Institute for Health Research Health Protection Research Unit on Modeling Methodology, Imperial College London; 2 Institute for Global Health, University College London; 3 Barts Health NHS Trust, London; 4 Infection Sciences, Viapath Analytics, Guy’s and St Thomas’ NHS Foundation Trust, London; 5 Public Health England, London; 6 Li Ka Shing Centre for Health Information and Discovery, Oxford University, United Kingdom

**Keywords:** HIV, men who have sex with men, phylodynamics, pre-exposure prophylaxis

## Abstract

**Background:**

The impact of HIV pre-exposure prophylaxis (PrEP) depends on infections averted by protecting vulnerable individuals as well as infections averted by preventing transmission by those who would have been infected if not receiving PrEP. Analysis of HIV phylogenies reveals risk factors for transmission, which we examine as potential criteria for allocating PrEP.

**Methods:**

We analyzed 6912 HIV-1 partial pol sequences from men who have sex with men (MSM) in the United Kingdom combined with global reference sequences and patient-level metadata. Population genetic models were developed that adjust for stage of infection, global migration of HIV lineages, and changing incidence of infection through time. Models were extended to simulate the effects of providing susceptible MSM with PrEP.

**Results:**

We found that young age <25 years confers higher risk of HIV transmission (relative risk = 2.52 [95% confidence interval, 2.32–2.73]) and that young MSM are more likely to transmit to one another than expected by chance. Simulated interventions indicate that 4-fold more infections can be averted over 5 years by focusing PrEP on young MSM.

**Conclusions:**

Concentrating PrEP doses on young individuals can avert more infections than random allocation.

## Teaser

Young men who have sex with men (MSM) in the United Kingdom have greater risk of infection and higher risk of transmission to other young MSM, which has implications for design of pre-exposure prophylaxis public health initiatives.

**(See the Editorial commentary by Baeten, on pages 1509–11.)**

The effectiveness of public health interventions (PHIs) to com bat human immunodeficiency virus (HIV), such as pre- exposure prophylaxis (PrEP) with antiretroviral medications (ARVs) depends on unknown variability of transmission risk in the infected population. The impact of PHIs can be enhanced if the intervention can be focused on patients with higher transmission risk, due to, for example, different risk behaviors or epidemiological settings [1]. HIV transmission risk is highly variable over time, over the course of infection, between risk groups, and geographically [2]. Biological, behavioral, and environmental factors shape individual HIV transmission risk in complex ways. Transmission probabilities per coital act depend on viral load [3], sexual positioning [4], male circumcision [5], and comorbidities [6]. Viral load is in turn mediated by the natural history of HIV infection, and many previous investigations have elucidated the role of early/acute HIV infection in enhancing transmission risk and increasing epidemic spread [7–9].

While the factors that shape transmission risk are understood qualitatively, it is challenging to obtain robust quantitative estimates of transmission probabilities or transmission risk. HIV genetic sequence data from routine drug resistance testing is one of the few sources of widely available observational data that are directly informative about HIV transmission patterns and transmission risk [10, 11]. Donor-recipient transmission pairs harbor virus that is genetically closely related compared to the population as a whole [12, 13]. At longer evolutionary time scales, populations or risk groups with higher transmission rates will tend to have a paraphyletic relationship with populations that are primarily recipients of infection. And over long periods of time, HIV genetic diversity is informative about the effective population size of the virus and epidemic growth rates [14, 15]. Genetic clustering of potential transmission pairs in large HIV sequence databases is a simple and scalable approach to characterizing transmission patterns [16], but genetic clustering is a highly unreliable proxy for transmission risk and inferences based on clusters are known to be biased by correlations with stage of infection at time of sampling [17–20]. In this study, we computed genetic clusters for a large sample of HIV-1 subtype B sequences in the United Kingdom and used these results to heuristically identify factors that may enhance individual-level transmission risk, with a particular focus on the role of young age on mediating HIV transmission in men who have sex with men (MSM). For selected variables with significant clustering associations, we performed a more robust phylodynamic analysis using formal phylogenetic and population genetic modeling [21]. Well-designed population genetic models can account for observed genetic diversity resulting from differential sampling effort over time [22] and between risk groups [23], and can account for nonlinear epidemic dynamics through time [24]. This analysis provided estimates of transmission risk ratios for selected biological and demographic covariates, which in turn informed a mathematical model of a PrEP intervention.

## METHODS

### Data

The UK HIV Drug Resistance Database contains more than 100000 sequences from more than 60000 patients at the end of 2015 (http://www.hivrdb.org.uk/). We extracted 6912 partial *pol* HIV-1 sequences and associated metadata (patient-level variables) that met the following criteria: (1) the sequence was subtype B determined using REGA [25]; (2) the sequence length was >1200 nucleotides; (3) the patient reported being MSM; and (4) the patient was treatment naive. We excluded all but the first sequence per patient if multiple sequences are available. We further restricted our analysis to samples that had a CD4 and/or recent infection testing algorithm (RITA) [26] result within 1 year of the sequence sample date in order to adjust for the effect of recency of infection on clustering and phylodynamic analyses. Sequences were collected between 1991 and the end of 2014 with 50% of samples collected after 2009.

To account for importation of HIV lineages, we added 1006 subtype B global reference sequences corresponding to unique sequences with highest similarity (using bitscore) after a BLAST search for each of the UK sequences. The BLAST database comprised 18544 global reference sequences (excluding UK sequences) obtained from Los Alamos HIV sequence database (https://www.hiv.lanl.gov, accessed October 2016). Drug resistance mutation sites as listed in the 2015 update from the International Antiviral Society-USA [27] were stripped from the alignment using the R package *big.phylo* (https://github.com/olli0601/big.phylo).

Multiple demographic and clinical covariates were available for each patient from Public Health England’s (PHE’s) HIV and AIDS Reporting System (HARS), which included persons diagnosed with HIV and seen for care. These data were linked to the UK Resistance database and included: (1) region of diagnosis corresponding to 12 reporting regions of PHE in the United Kingdom, (2) year of birth, (3) ethnicity, (4) CD4 counts, and (5) viral loads.

The work was conducted as part of the National Institute for Health Research Health Protection Research Unit (NIHR HPRU) at Imperial College London (Modelling Methodology), a partnership with PHE.

### Genetic Clustering

Heuristic genetic clustering analyses were carried out using threshold evolutionary distance, as described in [13]. Clusters were computed using thresholds of 0.5% and 1.5% distance using a TN93 substitution model, and sequence ambiguities were averaged when computing evolutionary distances between sequences. To identify variables that may be related to transmission risk, univariate logistic regression models were used to quantify the relative risk of clustering at different genetic distance thresholds. Multivariate logistic regression models including an indicator for early HIV infection (EHI) were used to adjust for upwards skew in frequency of clustering of recent HIV infections [20]. Young men who have sex with men (YMSM) were defined as patients with sequences sampled while the patient had an age less than 25 years, which corresponds to the bottom 10.7% of the age distribution. Both age at time of sampling and absolute age (corresponding to year of birth) may be important determinants of evolutionary history of an HIV lineage; however, we defined YMSM based on age at sampling because we anticipate this variable to have stronger association with recent transmission history of that lineage. The age threshold for defining YMSM is shared by recent studies [28] and was motivated by observed increasing odds of clustering with young age and the objective of identifying a relatively small risk group that would benefit from PrEP prioritization.

### Phylogenetic Analysis

Phylogenetic trees were estimated by maximum likelihood using ExaML and the R package *big.phylo* with a general time reversible model of nucleotide substitution and gamma distribution for rate heterogeneity among sites [29]. One hundred trees were reconstructed from bootstrap alignment. Three subtype G reference strains from Los Alamos database were used as outgroup for rooting the subtype-specific trees.

### Molecular Clock

We calculated root-to-tip distance from the phylogenetic tree and regressed distance by time of sampling. By iterations of Grubb’s algorithm [30] (https://CRAN.R-project.org/package=outliers), we identified and excluded 0.3% sequences as outliers in terms of divergence time and evolutionary rate. We applied least-square dating (LSD) algorithm [31] on rooted trees and sampling times to estimate the substitution rate and dates of ancestral nodes. To ensure accuracy of time-scaled phylogenies, the fast LSD method was compared to slower state of the art Bayesian methods (BEAST) [32] for a single clade using lineage-through-time statistics. Estimated lineages through time using LSD and BEAST are compared in the Supplementary information.

To facilitate computation with very large phylogenies, we divided the maximum likelihood tree into 21 disjoint clades defined by threshold time to most recent common ancestor (TMRCA). The threshold TMRCA was chosen such that the maximum number of sampled lineages in any clade was fewer than 1000 and the minimum clade size was 300. All clades had a TMRCA before 1980 and thus larger clades included both closely and distantly related sequences. Clades should not be confused with genetic distance clusters. Phylodynamic analyses were run in parallel on each clade.

### Phylodynamic Analysis

A structured coalescent model [33] was developed to estimate transmission risk ratios from the time-stamped HIV phylogeny while adjusting for stage of infection at time of sampling and differential sampling effort among young MSM and the remaining population. To adjust for stage of infection, we assigned each lineage to a CD4 stage described by Cori et al [34]. We defined the EHI stage to include both recent/acute infection and patients with high CD4 >500. Thus the EHI period is likely to encompass more than a year of the initial infectious period for most patients. Stage assignments were based on the CD4 result collected nearest in time to sequence sampling (maximum 1 year) as well as the RITA test result if available.

To model the dynamics of the number of infections and transmission rates within and between each deme, we developed a compartmental infectious disease model consisting of 7 ordinary differential equations, which described the number of infections in each of 3 stages of infection and 2 transmission risk levels corresponding to age group. The transmission rate was modeled as a product of 3 factors: (1) risk level according to a binary covariate such as being a young MSM; (2) stage of infection (EHI, chronic, or AIDS); and (3) secular trends in transmission rate. We modeled incidence of infection in each clade using a susceptible-infected-removed (SIR) model and estimated susceptible population size and transmission rate separately in each clade.

Importation of lineages into the United Kingdom was modeled using a single deme to represent the global HIV reservoir. The reservoir deme was designed to have exponentially growing effective population size with 2 free parameters which were estimated independently using a *skyspline* model [35]. Importation from the reservoir is modeled as a source-sink relationship with a constant rate per lineage. Once a lineage migrates from the reservoir, we assume that it may not circulate back to the reservoir.

Whereas transmission patterns between age groups may be highly nonrandom [36, 37] and transmissions are more likely between people in similar age groups, 2 additional parameters were estimated that describe the conditional probability of a YMSM transmitting to another YMSM and the probability of an older MSM (OMSM) transmitting to a YMSM.

Coalescent analyses were implemented using the *phydynR* R package (https://github.com/emvolz-phylodynamics/phydynR) and model parameters were estimated using maximum likelihood. Confidence intervals (CI) for transmission risk ratios of EHI and YMSM, age assortativity parameters, and exogenous lineage importation rates were computed using likelihood profiles.

A complete specification of the model equations, code, and estimation methodology is available in the Supplementary information.

### Predicting PrEP Intervention Effectiveness

We simulated a PrEP intervention based on provision of ARVs to approximately 15000 susceptible individuals who are vulnerable to HIV infection. This strategy is a modest scale-up of current plans to provide PrEP to 10000 eligible individuals over 3 years [38].

Two scenarios were considered in order to evaluate the benefit of prioritizing YMSM with higher risk of both infection and transmission. In the first scenario, PrEP was randomly allocated to all MSM irrespective of age, and in the second scenario, all PrEP was allocated to YMSM. Note that PrEP will not be allocated completely at random, and the first scenario is used as a benchmark rather than to model a likely outcome or standard of care. All simulations assumed 90% effectiveness in preventing infection. The population-level impact of PrEP depends on the proportion of susceptible individuals treated, and the number of susceptible MSM was extrapolated from recent HIV prevalence estimates and number diagnosed in the United Kingdom [39]. Given an estimated 45000 MSM diagnosed and undiagnosed living with HIV at the end of 2014 and HIV prevalence among MSM aged 15–44 between 4.1% and 5.8%, we infer there to be between 731000 and 1.05 million susceptible MSM. We therefore examined a range of proportions receiving PrEP of 1.4%–2.1% for all MSM or alternatively 13.4%–19.2% of YMSM. This simulation exercise did not account for self- medication with PrEP or potential differences in self-prophylaxis between age groups. The number of new HIV infections was simulated under both scenarios over a 5-year horizon by modifying the mathematical model fitted to HIV phylogenies and reducing transmission rates in proportion to the number of susceptibles receiving PrEP.

## RESULTS

Relative to older age groups, YMSM were more likely to be sampled with recent infection corresponding to higher CD4s and more frequent RITA-positive test results (Table 1). YMSM had significantly higher rates of EHI defined as CD4>500 or RITA positive test result (41% versus 23%, 2-sample binomial test). YMSM of subtype B were also more likely to reside outside of the London metropolitan area and less likely to be born outside of the United Kingdom. Relative to OMSM, YMSM were more likely to be genetically clustered with at least 1 other patient (31% versus 20%). More than 60% of both YMSM and OMSM clustered with at least 1 other patient using 1.5% threshold evolutionary distance and at 0.5% threshold distance about a quarter of patients clustered. Small but statistically significant associations were found between the odds of clustering at 0.5% and 1.5% evolutionary distance and EHI, age, and location of sampling. Patients sampled with EHI clustered slightly more in multivariate analyses (odds ratio [OR] = 1.14, 0.5% threshold) as do YMSM (OR = 1.09, 0.5% threshold).

**Table 1.** Demographic and Clinical Characteristics of YMSM and OMSM Included in the Analysis and all MSM in the Database

	YMSM/B (n = 706)	OMSM/B (n = 6206)	All MSM (n = 30711)
Year of birth (IQR)	1987(1984–1990)	1971(1964–1977)	1971(1964–1979)
CD4 (IQR)	**469(337–620**)	415(259–583)	420(260–590)
RITA+	34%	**25%**	28%
London	**39%**	**55%**	59%
White	84%	**88%**	85%
Immigrant	**19%**	**26%**	30%
Clustered (0.5%)	**31%**	20%	
Clustered (1.5%)	**77%**	62%	

YMSM and OMSM count only patients that meet all inclusion criteria. Statistical tests compare YMSM/B or OMSM/B and all MSM, except for the *clustered* outcome for which YMSM and OMSM are compared. Significance levels were determined using a 2-sample *t* test for continuous variables and Fishers exact test for binary variables.

Entries in bold have a *P* value < .001.

Abbreviations: IQR, interquartile range; OMSM, older men who have sex with men; YMSM, young men who have sex with men.

Coalescent-based phylodynamic analysis showed strong evidence of higher transmission risk for both EHI and YMSM. The transmission risk ratio of YMSM relative to all other MSM (OMSM) is 2.52 (95% CI, 2.32–2.73). The transmission risk ratio of EHI (CD4 >500 and/or RITA positive) relative to all other stages of infection is 3.70 (95% CI, 3.36–4.09). These represent independent effects, and YMSM with EHI were predicted to have the highest transmission risk.

**Figure 1.**
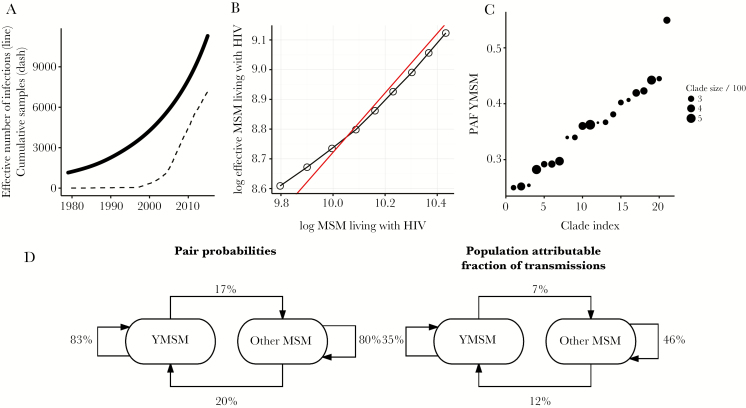
Effective number of infections through time, cumulative sequences sampled through time, and proportion of transmissions attributable to young men who have sex with men (YMSM) for 21 subtype B clades in UK MSM. *A*, Effective infections and cumulative samples combined for 21 clades. *B*, Number of MSM living with human immunodeficiency virus (HIV) estimated from surveillance data between 2004 and 2012 [39] versus phylodynamic estimates (log-transformed). A regression with slope constrained to 1 is shown in red. *C*, The estimated proportion of transmissions attributable to YMSM (age <25) for 21 clades and the number of samples in each clade. *D*, Estimated transmission patterns between YMSM and other MSM (OMSM). Left: The probability that a recipient is in each age group given that a donor is either YMSM or OMSM. Right: The proportion of all transmissions that are attributable to each pair of age groups. Abbreviation: PAF, population attributable fraction.

The phylodynamic analysis also revealed highly nonrandom transmission patterns by age. The probability that the recipient is YMSM given an infected donor in the OMSM risk group was 20.0% (95% CI, 17.7%–22.7%), which is roughly twice the proportion of the population that is YMSM (approximately 10% of MSM by definition). However, the probability that the recipient is YMSM given a YMSM donor was very much higher: 83.3% (95% CI, 78.4%–87.2%). Consequently, most YMSM were infected by other YMSM and not by older age groups. 75% of infections in YMSM were attributable to other YMSM, and 87% of infections in OMSM were attributable to other OMSM. Age assortativity and transmission patterns are summarized in Figure 1.

Finally, we estimated the average time that a HIV lineage has circulated in the United Kingdom prior to sampling. This was 27.6 years (95% CI, 26.6–28.75), suggesting that most subtype B infections in MSM are derived from introductions that occurred in the late 1980s [14].

The fitted population genetic model provided estimates of the number of effective infections through time (Figure 1 and Supplementary Figure S1) which approximately corresponds to the number of MSM living with HIV within the 21 clades included in this analysis. Note that these estimates were based on a subset of all HIV-1 genetic diversity and the absolute number of infections does not correspond to the total number of infections in the population. Nevertheless, the estimated rates of growth of MSM living with HIV are similar to estimates obtained by Public Health England based on surveillance data (Figure 1B and Supplementary Figure S2). We estimated that the 21 clades included samples from 60% of people living with HIV descended from the clades, the remainder not meeting inclusion criteria, not having sequences, or not being diagnosed in the United Kingdom. Note that the sample proportion is influenced by the fact that approximately 80% are diagnosed, not all diagnosed have a sequence in the database, and approximately 50% of lineages were excluded due to lack of adequate biomarkers or because they were collected from ART-experienced patients. There was substantial variation in the proportion of each clade that are YMSM, and the proportion of transmissions attributable to YMSM in each clade (Figure 1). The proportion of the clade that was YMSM was not significantly associated with growth rates of effective infections in each clade (F test *P* = .42).

**Figure 2.**
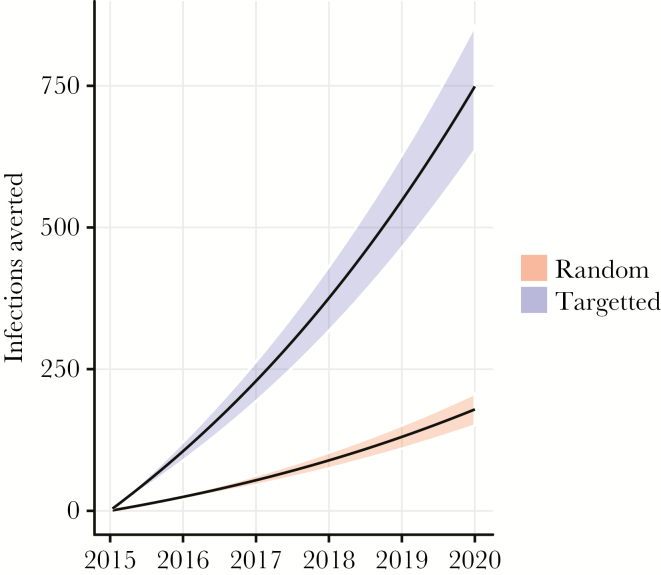
The number of infections averted over a 5-year horizon using a random allocation of pre-exposure prophylaxis (PrEP) or a targeted PrEP strategy focused on young men who have sex with men.

Simulated PrEP interventions based on the fitted population genetic model showed large gains from focusing PrEP on YMSM in comparison to random allocation to all MSM. Note that, in reality, PrEP would not be allocated randomly, and random allocation should be interpreted as a benchmark rather than a likely outcome or standard of care. Figure 2 shows the predicted cumulative infections averted by PrEP over 5 years if 15000 susceptible individuals were provided PrEP in 2015, which was the end point for sequence data included in this study. Simulations reflect not only direct impacts from preventing infections in treated individuals, but also indirect effects from preventing transmission by individuals who would have become infected without PrEP. We predicted that 749 (636–857) infections would be averted over 5 years if PrEP was focused on YMSM and that 179 (150–207) infections would be averted with random allocation. PrEP for YMSM averted 4.2 times as many infections over 5 years as random allocation.

## DISCUSSION

The combination of high levels of transmission and high assortativity amplifies incidence in YMSM and PrEP effectiveness in YMSM. PrEP in YMSM averts transmissions that would occur if YMSM were infected, and because YMSM are more likely to infect one another, subsequent generations of the epidemic process are further reduced. These transmission patterns yield higher incidence in YMSM, and it remains to corroborate observations based on phylodynamic analysis by estimating age-specific incidence in UK MSM from nongenetic surveillance data.

HIV genetic diversity in the United Kingdom showed a clear pattern consistent with higher risk of infection in YMSM and higher risk of transmission EHI. The effect of young age on transmission risk remained after controlling for higher rates of EHI among patients diagnosed at young age. YMSM have the hallmarks of a small high-risk core group [40], having both higher intrinsic transmission rates and preferential attachment within the risk group. YMSM show very high levels of assortative mixing. Most infections (75%) in YMSM arose via interaction with other YMSM despite comprising around 10% of the infected MSM population. These findings are consistent with previous reports of higher rates of HIV genetic clustering in YMSM [41, 42]. We find evidence for a modest net flow of transmissions from OMSM to YMSM which is in line with the findings of age-discordant clustering among young MSM found by Wolf et al [28]. These transmission patterns also differ from studies of genetic clustering in heterosexual populations, which have shown much greater age-discordancy within clusters that is hypothesized to arise from net flows of transmission from older males to younger females [43].

In reality, PrEP will not be allocated randomly, and age may provide one of many criteria for PrEP. Further studies are warranted to examine how age can be used in combination with other transmission and infection risk factors. Recent randomized clinical trials have examined the direct protective effects of PrEP in MSM but have not accounted for indirect transmission effects included in our simulations [44, 45]. Clinical trials, including studies conducted with UK MSM [46], have focused on individuals at higher risk of infection than MSM as a whole (eg, those with recent sexually transmitted infection [STI] testing history and condomless sex), so that estimates of infections averted are not directly comparable with our simulation results, which were constrained by the available data to focus on random allocation within age groups.

This study only examined subtype B sequences, which comprise the large majority of sequences among MSM in the United Kingdom. Less-prevalent subtypes have different demographic and clinical characteristics, notably CRF02AG which has higher proportions of recent African migrants (results not shown), and these results may not generalize to those clades. Compared to all sequences from MSM, the HIV lineages included in this analysis come from patients that are less likely to reside in London or be foreign born (Table 1).

Young age in MSM is a simple and easily identifiable proxy for transmission risk that may form one of many inputs into algorithms for prioritizing or promoting PrEP. Parameters estimated in this study may provide useful inputs into more detailed simulations for designing realistic PrEP interventions [47]. Access to PrEP and knowledge of PrEP may be lower in young MSM [48], providing further impetus to provide benefits for that group. YMSM can not be prioritized to the exclusion of other age groups, nor should age be the sole criterion used. Many risk groups were not considered in this phylogenetic analysis and it is important to have diverse as well as focused allocation of PrEP [49]. Other variables, such as recent STI testing history or self-reported risk exposures, are likely to also correlate highly with transmission risk, and it remains to examine such covariates within a phylodynamics framework.

## Supplementary Data

Supplementary materials are available at *The Journal of Infectious Diseases* online. Consisting of data provided by the authors to benefit the reader, the posted materials are not copyedited and are the sole responsibility of the authors, so questions or comments should be addressed to the corresponding author.

Supplementary Material 1

Supplementary Material 2

## Notes

***Acknowledgments.*** This research was made possible by the Steering Committee and Coordinating Centre of the UK Drug Resistance Database the Centres contributing data to the UK Drug Resistance Database. A full list of contributors is provided in the online Supporting material.

***Funding.*** This work was supported by the UK Health Protection Research Units in Modeling Methodology and Sexually Transmitted Infections and by the National Institutes of Health (grant number R01AI087520).

***Potential conflicts of interest.*** All authors: No reported conflicts of interest. All authors have submitted the ICMJE Form for Disclosure of Potential Conflicts of Interest. Conflicts that the editors consider relevant to the content of the manuscript have been disclosed.

Presented in part: HIV Dynamics and Evolution conference, Skye, United Kingdom, 25 May 2017.
