# Supplementary material for: Molecular Epidemiology of HIV-1 Subtype B Reveals Heterogeneous Transmission Risk: Implications for Intervention and Control
Source: J Infect Dis. 2018 Feb 26;217(10):1522–9. doi: 10.1093/infdis/jiy044 (PMC5913615; doi:10.1093/infdis/jiy044)
Supplement: Supplementary Material 1 [file jiy044_suppl_supplementary-material_1.docx]

## Acknowledgments

Steering Committee of the UK Drug Resistance Database: David Asboe, Anton Pozniak (Chelsea & Westminster Hospital, London); Patricia Cane (Public Health England, Porton Down); David Chadwick (South Tees Hospitals NHS Trust, Middlesbrough); Duncan Churchill (Brighton and Sussex University Hospitals NHS Trust); Duncan Clark (Barts Health NHS Trust, London); Simon Collins (HIV i-Base, London); Valerie Delpech (National Infection Service, Public Health England); Samuel Douthwaite (Guy’s and St. Thomas’ NHS Foundation Trust, London); David Dunn, Esther Fearnhill, Kholoud Porter, Anna Tostevin, Ellen White, Oliver Stirrup (Research Department of Infection and Population Health, UCL); Christophe Fraser (University of Oxford); Anna Maria Geretti (Institute of Infection and Global Health, University of Liverpool); Rory Gunson (Gartnavel General Hospital, Glasgow); Antony Hale (Leeds Teaching Hospitals NHS Trust); Stéphane Hué (London School of Hygiene and Tropical Medicine); Steve Kaye (Imperial College, London); Linda Lazarus (Expert Advisory Group on AIDS Secretariat, Public Health England); Andrew Leigh-Brown (University of Edinburgh); Tamyo Mbisa (National Infection Service, Public Health England); Nicola Mackie (Imperial NHS Trust, London); Samuel Moses (King’s College Hospital, London); Chloe Orkin (Barts Health NHS Trust, London); Eleni Nastouli, Deenan Pillay, Andrew Phillips, Caroline Sabin (University College London, London); Erasmus Smit (Public Health England, Birmingham Heartlands Hospital); Kate Templeton (Royal Infirmary of Edinburgh); Peter Tilston (Manchester Royal Infirmary); Ian Williams (Mortimer Market Centre, London); Hongyi Zhang (Addenbrooke’s Hospital, Cambridge).

Coordinating Centre of the UK Drug Resistance Database: Research Department of Infection and Population Health, UCL (David Dunn, Keith Fairbrother, Esther Fearnhill, Kholoud Porter, Anna Tostevin, Ellen White, Oliver Stirrup)

Centres contributing data to the UK Drug Resistance Database: Clinical Microbiology and Public Health Laboratory, Addenbrooke’s Hospital, Cambridge (Jane Greatorex); Guy’s and St Thomas’ NHS Foundation Trust, London (Siobhan O’Shea, Jane Mullen); PHE – Public Health Laboratory, Birmingham Heartlands Hospital, Birmingham (Erasmus Smit); Antiviral Unit, National Infection Service, Public Health England, London (Tamyo Mbisa); Imperial College Health NHS Trust, London (Alison Cox); King’s College Hospital, London (Richard Tandy); Medical Microbiology Laboratory, Leeds Teaching Hospitals NHS Trust (Tracy Fawcett); Specialist Virology Centre, Liverpool (Mark Hopkins); Department of Clinical Virology, Manchester Royal Infirmary, Manchester (Peter Tilston); Department of Virology, Royal Free Hospital, London (Clare Booth, Ana Garcia-Diaz); Edinburgh Specialist Virology Centre, Royal Infirmary of Edinburgh (Lynne Renwick); Department of Infection & Tropical Medicine, Royal Victoria Infirmary, Newcastle (Matthias L Schmid, Brendan Payne); South Tees Hospitals NHS Trust, Middlesbrough (David Chadwick); Department of Virology, Barts Health NHS Trust, London (Duncan Clark, Jonathan Hubb); Molecular Diagnostic Unit, Imperial College, London (Steve Kaye); University College London Hospitals (Stuart Kirk); West of Scotland Specialist Virology Laboratory, Gartnavel, Glasgow (Rory Gunson, Amanda Bradley-Stewart).
