# Supplementary material for: Molecular Epidemiology of HIV-1 Subtype B Reveals Heterogeneous Transmission Risk: Implications for Intervention and Control
Source: J Infect Dis. 2018 Feb 26;217(10):1522–9. doi: 10.1093/infdis/jiy044 (PMC5913615; doi:10.1093/infdis/jiy044)
Supplement: Supplementary Material 2 [file jiy044_suppl_supplementary-material_2.pdf]

Supporting information

Mathematical model

Infectious disease dynamics are modelled separately for each clade. The following system of equations were used to simulate epidemic trajectories for each clade; some parameters were estimated specifically for each clade, while other parameters had shared values for all clades. First we will specify the equations and then describe which parameters are clade-specific and the model fitting methodology. A graphical representation of the model is shown in supporting Figure S3 Fig.

$I_1(t), I_2(t)$, and $I_3(t)$ denote the number of OMSM infected at time t in EHI (stage 1), chronic (stage 2) or late infection including AIDS (stage 3). Similarly, $J_1(t), J_2(t)$, and $J_3(t)$ denote the same quantities for YMSM. The variable $Y(t)$ represents the global HIV reservoir and is a source of imported lineages into the UK. These are the only dynamic variables and the time argument t will be dropped from subsequent equations.

Stage definitions were based on recent analyses by Cori et al. which showed how to realistically model the natural history of HIV infection with compartmental continuous time Markov Processes [1]. Stage 1 corresponds to CD4 exceeding 500 at time of sampling and/or a RITA positive test result. Stage 2 corresponds to CD4 between 200 and 500 and stage 3 corresponds to CD4 < 200. Adapting the analysis by Cori et al., we define the following rates of disease progression:

- $\gamma_1 = 0.34$: Per capita rate (1/year) of progressing from stage 1 to 2

- $\gamma_2 = 0.13$: Per capita rate(1/year) of progressing from stage 2 to 3
- $\gamma_3 = 0.20$: Per capita rate(1/year) of progressing from stage 3 to death and/or effective viral suppression with treatment

OMSM experience natural mortality with per capita rate $\mu = 0.032$ per person per year. This value was chosen to correspond to a life expectancy of 75 years and was based on the average age at time of sampling for OMSM. YMSM age naturally into the OMSM category at the rate $\phi = 0.25$ per person per year. This value was based on the age threshold for YMSM and the average age among patients sampled in the YMSM category.

With the above definitions of the aging and natural history parameters, we can specify the following ordinary differential equations for chronic and AIDS infections:

$$\dot{I}_2 = \gamma_1 I_1 - (\gamma_2 + \mu) I_2 + \phi J_2 \quad (1)$$

$$\dot{I}_3 = \gamma_2 I_2 - (\gamma_3 + \mu) I_3 + \phi J_3 \quad (2)$$

$$\dot{J}_2 = \gamma_1 J_1 - (\gamma_2 + \phi) J_2 \quad (3)$$

$$\dot{J}_3 = \gamma_2 J_2 - (\gamma_3 + \phi) J_3 \quad (4)$$

Note that $\dot{x}$ represents the derivative of x with respect to time.

The equations for numbers with EHI will depend on the transmission process which we now describe. Define $\iota(t)$ to be the total rate of new infections at time t. This will be modeled using a simple SIRS model:

$$\iota(t) = \beta(I + J)S/N$$

where

- S is the effective number susceptible

- β is the mean per capita transmission rate
- $I = I_1 + I_2 + I_3$ is the total number of infected OMSM
- $J = J_1 + J_2 + J_3$ is the total number of infected YMSM
- $N = S + I + J$ is the total population sizes

Given a transmission event, several parameters are used to model the probability that the source is a YMSM or OMSM and the stage of infection, and the probability that the recipient is YMSM or OMSM:

- W_{ehi} is the transmission risk ratio for EHI (stage 1) relative to stages 2 and 3
- W_{ymsm} is the transmission risk ratio for YMSM relative to OMSM
- p_{yy} is the probability that the recipient of infection is YMSM given a YMSM donor
- p_{oy} is the probability that the recipient of infection is YMSM given a OMSM donor

The probability that the donor is each of the following demes is proportional to the following quantities

- $J_1 : W_{ehi} W_{ymsm} J_1$
- $J_2 : W_{ymsm} J_2$
- $J_3 : W_{ymsm} J_3$
- $I_1 : W_{ehi} I_1$
- $I_2 : I_2$

- $I_3 : I_3$

The variable C denotes the sum of weights:

$$C = W_{ehi}W_{ymsm}J_1 + W_{ymsm}J_2 + W_{ymsm}J_3 + W_{ehi}I_1 + I_2 + I_3$$

So we define the following probabilities that the donor is in each deme

- $q_{J1} : W_{ehi}W_{ymsm}J_1/C$
- $q_{J2} : W_{ymsm}J_2/C$
- $q_{J3} : W_{ymsm}J_3/C$
- $q_{I1} : W_{ehi}I_1/C$
- $q_{I2} : I_2/C$
- $q_{I3} : I_3/C$

With the above definitions in place, the dynamics of EHI for YMSM and OMSM are specified by the following equations:

$$\dot{J}_1 = \iota(t)(p_{yy}(q_{J1} + q_{J2} + q_{J3}) + p_{oy}(q_{I1} + q_{I2} + q_{I3})) - (\gamma_1 + \phi)J_1 \tag{5}$$

$$\dot{I}_1 = \iota(t)((1 - p_{yy})(q_{J1} + q_{J2} + q_{J3}) + (1 - p_{oy})(q_{I1} + q_{I2} + q_{I3})) - (\gamma_1 + \mu)J_1 \tag{6}$$

Initial conditions are modeled using a single parameter for the number of infections at $t = 1979$ which are uniformly distributed across demes.

Reservoir dynamics $Y(t)$ were estimated separately using the *skyspline* method [2]. Importation is modelled as a constant rate r per lineage. Importation is sufficiently rare that model assumes it does not substantially impact deme sizes I or J.

The PrEP simulations were based on a reduction in the effective proportion of the population that is susceptible. The transmission rate

between each pair of demes was scaled downwards in proportion to the number of individuals in the recipient deme that receive PrEP. The proportions in each deme receiving PrEP were based on national prevalence estimates and the number of observed diagnosed up to the end of 2015 as described in the main text.

Code and scripts for simulating this model are included as supporting files.

Statistical methods

Maximum likelihood estimates were obtained for the following parameters estimated independently for each clade:

- β_i for subtype B clade $i = 1 \cdots 21$. This is the per capita transmission rate as described in the model above.

- N_i for subtype B clade $i = 1 \cdots 21$. The effective initial susceptible population size for each clade.

- $I_0^{(i)}$ for subtype B clade $i = 1 \cdots 21$. The initial conditions for each clade; this is the total number infected at $t = 1979$.

Additionally, the following parameters were shared between all clades:

- W_{ehi}: Transmission risk ratio for EHI

- W_{ymsm}: Transmission risk ratio for YMSM

- r: The lineage importation rate from the global HIV reservoir into the UK

- p_{yy}: The probability that the recipient of a YMSM donor is also YMSM

- p_{oy}: The probability that the recipient of a OMSM donor is YMSM

We denote the two sets of parameters using the vectors

$$\theta_i = (\beta_i, N_i, I_0^{(i)}) \quad i = 1 \cdots 21 \tag{7}$$

$$\zeta = (W_{ehi}, W_{ymsm}, r, p_{yy}, p_{oy}) \tag{8}$$

Likelihoods of epidemic parameters given time-scaled phylogenies were computed using the *phydynR* R package (`http://github.com/phydynR`, obtained April 6, 2017), which uses a structured coalescent model derived from the methods described in [3]. Likelihoods were computed independently for each clade. Only phylogenetic nodes 25 years before present were included in likelihood calculations. Because of the large number of free parameters and large sample size ($n > 6,000$ including all clades), an iterative algorithm was used to compute MLEs of all parameters:

1. Starting conditions were proposed for all parameters
2. For iteration $k = 1 : m$
   (a) For each clade $i = 1 \cdots 21$ MLEs were computed: $\hat{\theta}_i^{(k+1)} | \hat{\zeta}^k$
   (b) By summing likelihoods for all clades, MLEs were computed: $\hat{\zeta}^{(k+1)} | \hat{\theta}_i^{(k+1)}$
3. The procedure is terminated when convergence is observed in the likelihood and MLE of all parameters, that is when the improvement in log likelihood was less than 0.01 units.

Note that both steps of the above MLE procedure made use of parallel computation of likelihoods for each clade, which greatly facilitated estimation with large sample size. All MLE's were computing using the

simplex optimisation method in R (*optim*) [4, 5]. Convergence of likelihoods and parameters is shown in supporting Figure S4 Fig.

Likelihood profiles

Confidence intervals were computed using likelihood profiling techniques combined with a smoothing spline approximation to the likelihood function in the vicinity of the MLE [6]. First, the Hessian of the likelihood function was computing using *optim* in R. This was used to derive an approximate variance covariance matrix of parameters in ζ. For each parameter, a region was searched within 5 approximate standard deviations of the MLE. An Akima spline [7] was used to approximate the likelihood function based on profiled points. Likelihood profiles are shown in supporting Figure S5 Fig.

Molecular clock comparisons

As a check on the quality of the molecular clock phylogenetic analysis using RaxML and least-squares-dating [8], we conducted a Bayesian phylogenetic analysis of a subset of sequences using BEAST 1.8 [9]. Bayesian relaxed clock methods have been well validated, but are computationally intensive and can not scale to the sample size of the UK resistance database. To pick a subset of sequences for comparison, we used threshold genetic distance clustering and a large threshold of 2% TN93 distance. We selected a cluster of $n = 153$ sequences because it had a large range of sample dates and a moderate sample size.

The BEAST method was applied to this cluster with the following parameters

- GTR + Gamma + I substitution model

- Uncorrelated lognormal relaxed clock
- Skyride coalescent prior [10]
- We ran the algorithm for 150 million iterations, sampling every 10 thousand iterations

One hundred trees were sampled from the BEAST posterior using a burn-in of 33%. We assessed convergence of the Bayesian MCMC method using Tracer [11]. The minimum effective sample size of all parameters was 322. A mean substitution rate of 1.6 per thousand bases per year was estimated, which was in line with the estimated LSD clock rate.

A comparison of the BEAST posterior trees and 100 trees from the LSD bootstrap distribution is shown in supporting Figure S6 Fig. The lineages through time estimated from BEAST and LSD are highly similar for most of the evolutionary history of this cluster, however noticeable deviation occurs before 20 years of the most recent sample. The coalescent inference described above is based on 25 years of evolutionary history, indicating that results are likely to be similar if based on Bayesian relaxed clock phylogenetic methods.

S1 Fig. Estimated effective number of infections in each of 21 clades. Panel B shows the effective number infected divided by the total number of patients sampled in each clade.

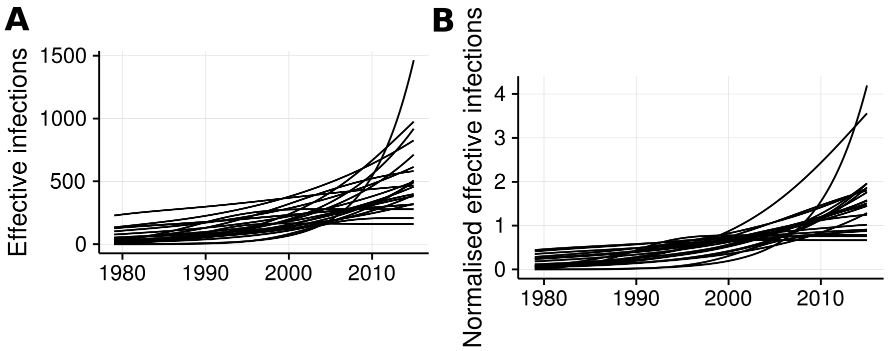

S2 Fig. Estimates of the number of people living with HIV 1995-2012 in the UK and in all risk groups based on surveillance data and estimates of the number of infected MSM corresponding to the subset of 21 clades using phylodynamic analysis.

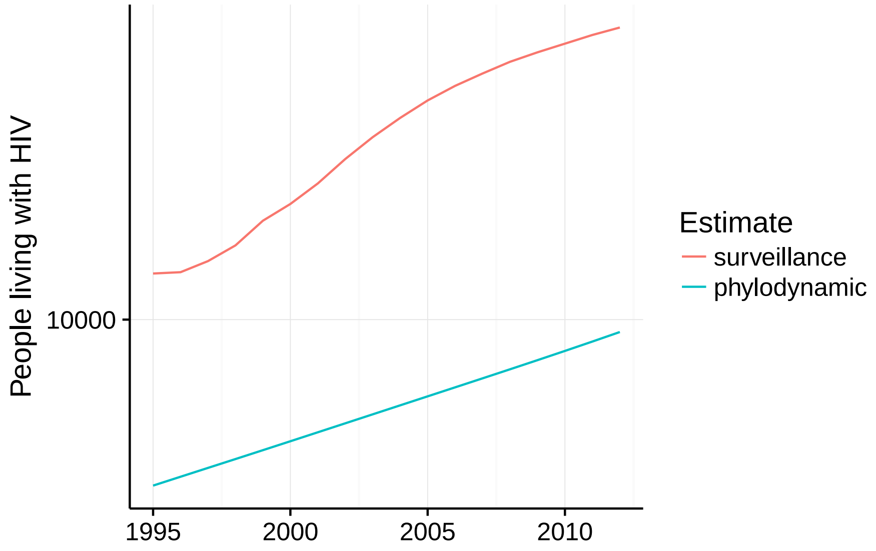

S3 Fig. Diagram representing structure of mathematical model. Circles represent states of infected individuals and arrows represent transitions between states.

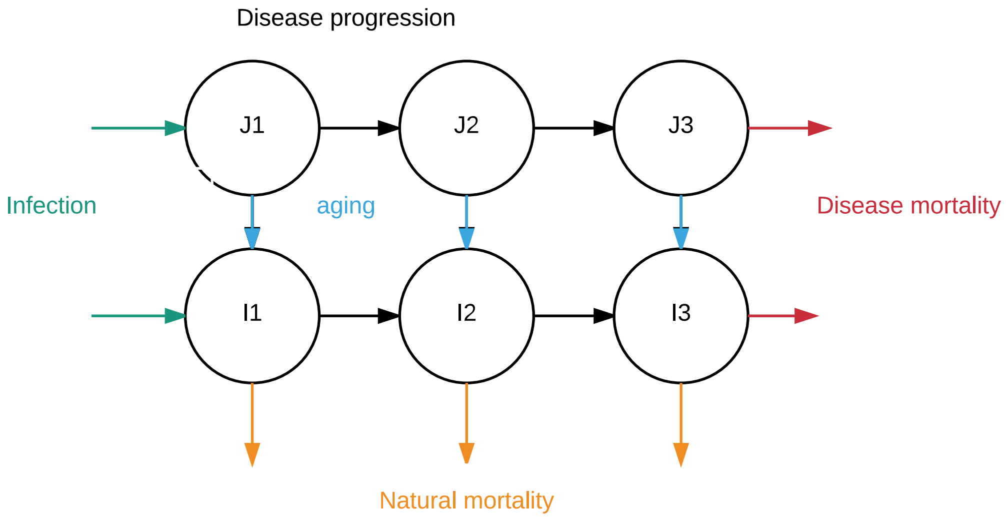

S4 Fig. Likelihood and estimated parameter values at each iteration of the fitting algorithm.

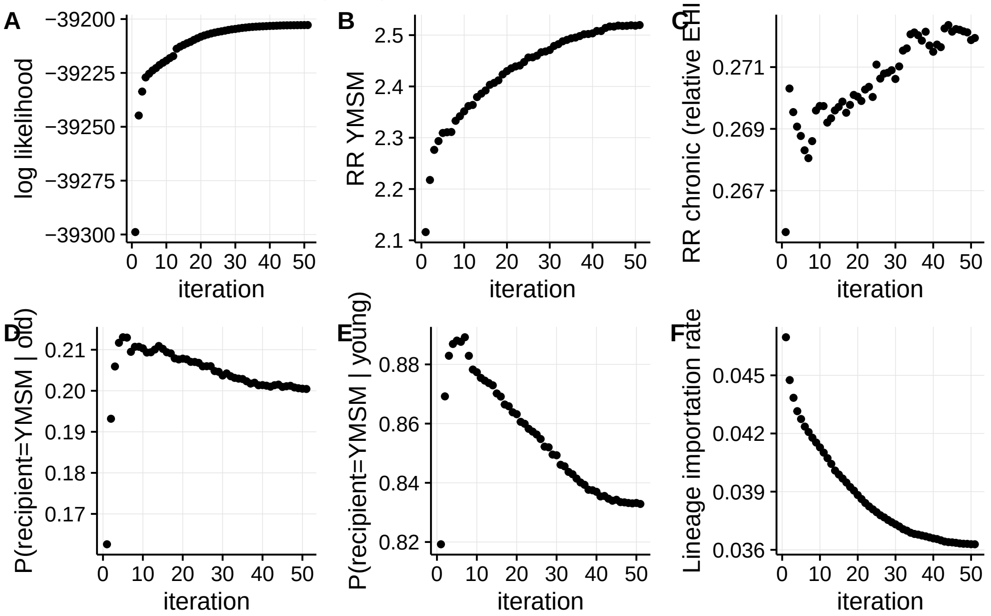

S5 Fig. Likelihood profiles for model parameters. Black line shows a smoothing spline. Red vertical lines show 95% confidence intervals inferred from each profile. $p(Y|O)$ in panel B refers to the probability that a recipient is YMSM given a donor that is OMSM.

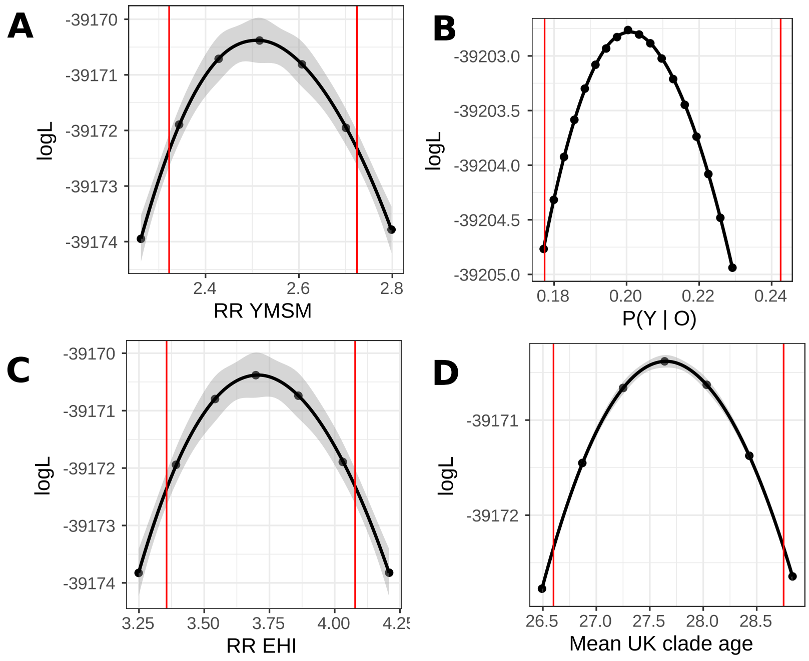

S6 Fig. Comparison of lineages through time and phylogenetic node times (TMRCAs) estimated using BEAST and LSD. A. Lineages through time for 100 trees from the BEAST posterior and 100 trees from the LSD bootstrap distribution. B. Comparison of the distribution of internal node times estimated by BEAST and LSD.

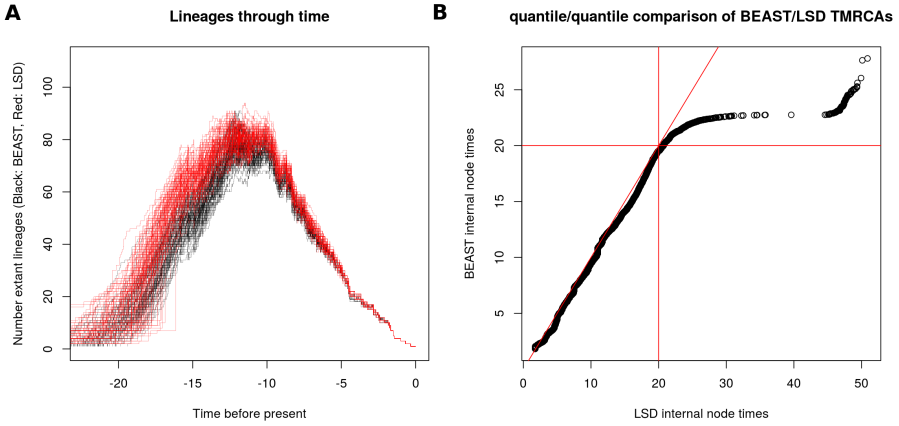

References

1. Cori A, Pickles M, van Sighem A, Gras L, Bezemer D, Reiss P, et al. CD4+ cell dynamics in untreated HIV-1 infection: overall rates, and effects of age, viral load, sex and calendar time. AIDS. 2015;29(18):2435–2446.

2. Volz E, Romero-Severson E, Leitner T. Phylodynamic inference across epidemic scales. Molecular Biology and Evolution. 2017;.

3. Volz EM. Complex population dynamics and the coalescent under neutrality. Genetics. 2012;190(1):187–201.

4. Nelder JA, Mead R. A simplex method for function minimization. The computer journal. 1965;7(4):308–313.

5. R Core Team. R: A Language and Environment for Statistical Computing. Vienna, Austria; 2017. Available from: `https://www.R-project.org/`.

6. Ionides EL, Breto C, Park J, Smith RA, King AA. Monte Carlo profile confidence intervals. arXiv preprint arXiv:161202710. 2016;.

7. Akima H. A method of univariate interpolation that has the accuracy of a third-degree polynomial. ACM Transactions on Mathematical Software (TOMS). 1991;17(3):341–366.

8. To TH, Jung M, Lycett S, Gascuel O. Fast dating using least-squares criteria and algorithms. Systematic Biology. 2015;p. syv068.

9. Drummond AJ, Rambaut A. BEAST: Bayesian evolutionary analysis by sampling trees. BMC Evolutionary Biology. 2007;7(1):214.

10. Minin VN, Bloomquist EW, Suchard MA. Smooth skyride through a rough skyline: Bayesian coalescent-based inference of population dynamics. Molecular Biology and Evolution. 2008;25(7):1459–1471.

11. Rambaut A, Suchard MA, Xie D, Drummond AJ. Tracer v1. 6. 2014; 2015.
